# Dynamic CT perfusion image data compression for efficient parallel processing

**DOI:** 10.1007/s11517-015-1331-6

**Published:** 2015-06-24

**Authors:** Renan Sales Barros, Silvia Delgado Olabarriaga, Jordi Borst, Marianne A. A. van Walderveen, Jorrit S. Posthuma, Geert J. Streekstra, Marcel van Herk, Charles B. L. M. Majoie, Henk A. Marquering

**Affiliations:** Biomedical Engineering and Physics, Academic Medical Center, University of Amsterdam, Location L0, Meibergdreef 15, 1105 AZ Amsterdam, The Netherlands; Department of Clinical Epidemiology, Biostatistics and Bioinformatics, Academic Medical Center, University of Amsterdam, Location B0, Meibergdreef 9, 1105 AZ Amsterdam, The Netherlands; Department of Radiology, Academic Medical Center, University of Amsterdam, Location B0, Meibergdreef 9, 1105 AZ Amsterdam, The Netherlands; Department of Radiology, Leiden University Medical Center, Albinusdreef 2, 2333 ZA Leiden, The Netherlands; Department of Radiation Oncology, The Netherlands Cancer Institute, Plesmanlaan 121, 1066 CX Amsterdam, The Netherlands

**Keywords:** Acute care, CT perfusion, GPU, Lossless compression, Parallel processing

## Abstract

The increasing size of medical imaging data, in particular time series such as CT perfusion (CTP), requires new and fast approaches to deliver timely results for acute care. Cloud architectures based on graphics processing units (GPUs) can provide the processing capacity required for delivering fast results. However, the size of CTP datasets makes transfers to cloud infrastructures time-consuming and therefore not suitable in acute situations. To reduce this transfer time, this work proposes a fast and lossless compression algorithm for CTP data. The algorithm exploits redundancies in the temporal dimension and keeps random read-only access to the image elements directly from the compressed data on the GPU. To the best of our knowledge, this is the first work to present a GPU-ready method for medical image compression with random access to the image elements from the compressed data.

## Introduction

CT perfusion (CTP) imaging is used as a diagnostic tool for initial evaluation of patients suffering from acute stroke [1]. CTP images are acquired by dynamically tracking the passage of a contrast agent through the cerebral blood vessels and tissue [2]. Analysis of CTP data enables the assessment of the severity of the damages caused by stroke. This information can be used to choose the most adequate treatment for the patient [3]. Currently, CTP datasets can be as large as 3.76 GB, and when dealing with this amount of data, traditional processing methods are slow and delay the acute care. Also, these traditional methods are expensive because of the costs of purchase and maintenance of dedicated software and hardware for image processing.

Cloud architectures have emerged as a cost-effective alternative for medical image processing. Cloud-based solutions make remote on-demand image processing services available for wide use in medical practice. To provide high-performance processing, cloud architectures can make use of graphics processing units (GPUs), which are designed for very efficient parallel processing of large amounts of data. GPUs were demonstrated being capable of considerably speeding up medical image processing applications [4]. Nowadays CPUs are also capable of parallel processing. However, CPUs are designed for general purpose processing, and because of that, the processing power of a GPU can be superior to the processing power of a CPU in several applications. GPUs are used in several common image processing tasks such as filtering and rendering. Thus, it is feasible to assume that the processing of CTP image data can also take advantage of GPU-based architectures. However, to benefit from the GPU computational power, algorithms need to be adapted or developed from scratch.

The size of CTP data poses challenges for their processing on GPU and on cloud infrastructures. The transfer of CTP data to cloud architectures can be time-consuming, which may limit the suitability of cloud applications for dealing with acute patients. In addition, to perform GPU computation, a host application is required, and the CTP data also need to be transferred from the host memory to the GPU memory. The time spent on the transfers from host to GPU has a considerable impact on the overall processing time. In short, due to the large size of CTP datasets, the time to transfer the image data limits its application for remote processing in acute care scenarios.

Data compression techniques can be used to reduce the CTP dataset size and speed up its transfer to the cloud and to the GPU memory. Since time is critical in acute situations, the time required to compress, decompress, and transfer the compressed data should not be larger than the time required to transfer the uncompressed data. Another important constraint is that, in clinical care applications, the compression technique must be lossless because no information can be removed or modified due to legal regulations.

**Fig. 1:**
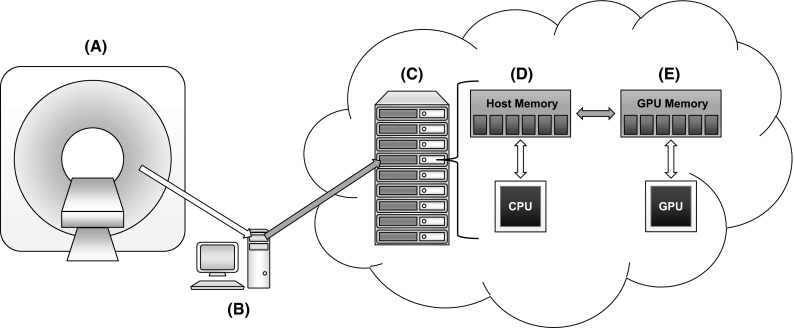
CTP data processing pipeline in a GPU-based cloud infrastructure: the CTP data are produced at the scanner (*A*), compressed in a terminal (*B*), sent to the GPU-based cloud infrastructure (*C*). While being processed, the CTP data can be transferred several times between host application memory (*D*) and GPU memory (*E*)

The time required to execute the complete CTP data pipeline depends on scanner acquisition, data reconstruction, preprocessing, etc. Several aspects of this pipeline are strictly determined by scanner manufacturers. Figure 1 illustrates which pipeline stages (dark arrows) of the CTP processing in a GPU-based cloud infrastructure are affected by our compression method. Initially, the CTP data are produced at the scanner (A). After that, the CTP data must be compressed in a terminal (B) before the transfer to the GPU-based cloud infrastructure (C). While the CTP data are processed in the cloud infrastructure, several data transfers between host application memory (D) and GPU memory (E) can be required.

Ideally, the compression must be done in a machine capable of GPU processing. However, the compression can be executed in different computing devices such single-core CPUs and many-core CPUs.

The main goal of our compression technique is to reduce the data size for faster transfer and faster GPU processing on cloud architectures. To achieve this, we introduce a fast and lossless compression technique that not only speeds up the transfer of dynamic CTP data to cloud architectures, but also facilitates their parallel processing on GPUs. This technique presents a compression time suitable for acute care situations and produces compressed data that can be processed on a GPU requiring no decompression of the entire CTP dataset. In our technique, intensities of an arbitrary voxel are retrieved from the compressed data using a fixed amount of instructions independent of the input value or size. This means that, in terms of computational complexity, determining the intensity value of a voxel is a constant-time procedure (i.e., checking if a number is odd or even, checking a constant size lookup table), which is the fastest class of algorithms with computational complexity classified as *O*(1). To the best of our knowledge, this is the first work to present a lossless method for medical image compression with direct access to the image elements from the compressed data.

## Methods

This section describes the characteristics of the CTP data, presents our compression technique, and discusses the relevant aspects that need to be considered during its implementation according to the targeted platform. Subsequently, the configuration of the experiments used to evaluate our compression technique is described.

### Characteristics of CTP data

The datasets used in this study consist of 20 dynamic whole-brain volumes from actual stroke patients. The scans have 320 slices of 512 × 512 voxels with 16 bits/voxel, and each acquisition has 24 time steps. The patients were scanned as part of a Dutch multicenter randomized trial [5]. Approval of the medical ethical committee was obtained. All patients or legal representatives signed informed consent. The volumes are acquired approximately every 2.5 s during the first 35 s, followed by a scan every 5 s until 60 s. Subsequently, five volumes are scanned with a 30-s interval. The size of each volume is 160 MB, and thus the complete dataset has 3840 MB of data that need to be quickly processed for an initial evaluation of the patient condition. Sometimes, an additional CTP dataset is produced to evaluate the treatment progress after around 24 h, resulting in up to 7.5 GB of data per patient. All the image data are saved according to the digital imaging and communications in medicine (DICOM) standard.

**Fig. 2:**
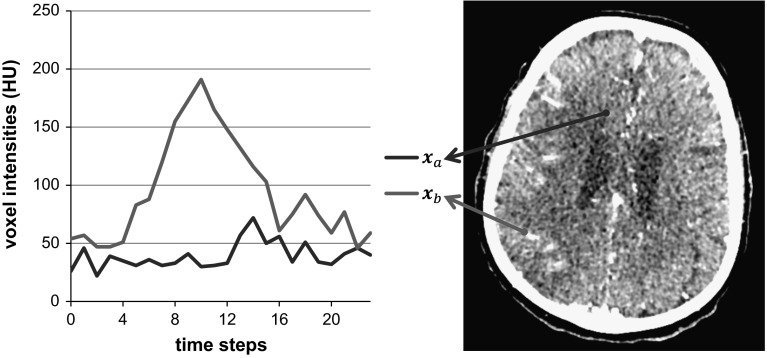
Sample slice of CTP data at the time step 12, and the intensity values of the voxels at ${{\mathbf{x}}_a}$ and ${{\mathbf{x}}_b}$ over time. The intensities values at ${{\mathbf{x}}_a}$ are not strongly affected by the contrast agent, and the intensities values at ${{\mathbf{x}}_b}$ are strongly affected by contrast agent

Every dataset can be described as $I({\mathbf{x}},t)$, which represents the image intensity at position ***x*** at time *t*. The inflow and outflow of contrast agent can be observed in all the brain tissue. However, the intensity values in the largest part of the brain tissue are expected to vary little over time. To illustrate this characteristic of the data, Fig. 2 shows the intensities at ${{\mathbf{x}}_a}$ and ${{\mathbf{x}}_b}$ along time. The intensities at ${{\mathbf{x}}_a}$ are not strongly affected by the contrast agent. On the other hand, the intensities at ${{\mathbf{x}}_b}$ are strongly affected by the inflow and outflow of contrast agent.

Voxel intensities in CT imaging are generally represented using 16 bits. However, the range of voxel values over time is smaller than the range that can be represented by 16 bits. Therefore, fewer bits can be used to represent exactly the same information by storing the variation of these intensities instead of their absolute values. This characteristic is illustrated by using the intensities at ${{\mathbf{x}}_b}$ as an example. These intensities vary between 46 and 191 HU, so only eight bits are required to represent them ($\left\lceil {{{\log }_2}(191 - 46 + 1)} \right\rceil = 8$). For the voxel at ${{\mathbf{x}}_a}$, a better compression can be obtained because only six bits are required ($\left\lceil {{{\log }_2}(72 - 22 + 1)} \right\rceil = 6$), which represents a compression ratio of 2.6 compared with the original representation using 16 bits.

**Fig. 3:**
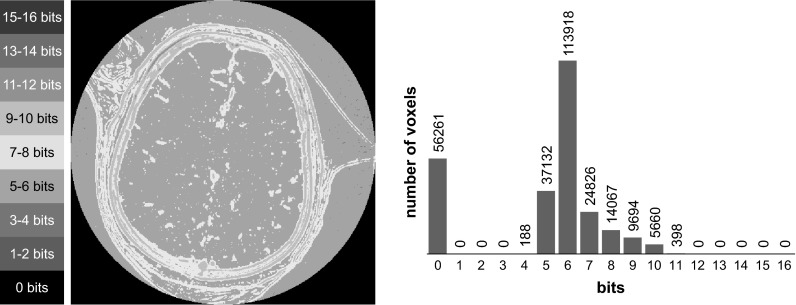
Number of bits required to represent the variation of voxel intensities over time in the selected slice. The effect of motion artifacts is visible, and for this reason, a higher amount of bits is required to represent the area around the skull. Nevertheless, this higher amount of bits (9–11 bits) is considerably smaller than the original 16 bits that are used by the uncompressed data. Furthermore, the motion affects only a small portion of the image. Only 6 % of the voxels require more than eight bits to represent their intensities variation over time

As observed in Fig. 3, only 6 % of the voxels in that slice require more than eight bits to represent their intensities variation over time, and a maximum of 11 bits is required to represent this variation.

The effect of motion artifacts is apparent in Fig. 3, and for this reason, a higher amount of bits is required to encode the area around the skull. However, this higher amount of bits (between 9 and 11 bits) is still considerably smaller than 16 bits, which are required for the uncompressed image. Furthermore, the motion affects only a small portion of the image. In general, when motion is present, there is mainly overlapping of brain tissue with similar intensity values, which does not result in a higher amount of bits for encoding the voxel intensities over time. In short, Fig. 3 illustrates that, due to the characteristics of the CTP data, the number of voxels that have a large intensities variation over time is rather small. This indicates that the temporal dimension of the CTP data is a substantial source of redundancies that can be exploited for compression purposes.

### Compression algorithm

Our compression technique exploits the time redundancy explained above. Let $I({\mathbf{x}},t)$ represent the uncompressed image intensity, where ***x*** indicates a 3D coordinate and *t* indicates a time step between 0 and *n* − 1. In compressed form, the image is represented as

$$I({\mathbf{x}},t) = C({\mathbf{x}}) + \varDelta ({\mathbf{x}},t)$$

with

$$\hbox{min} {V_x} \leqslant C({\mathbf{x}}) \leqslant \hbox{max} {V_x}$$

and

$${V_x} = \{ I({\mathbf{x}},{t_0}),I({\mathbf{x}},{t_1}), \ldots ,I({\mathbf{x}},{t_{n - 1}})\} .$$

For simplicity, we use

$$C({\mathbf{x}}) = \hbox{min} {V_x}$$

The set of values *D*_*x*_ given by

$${D_x} = \{ \varDelta ({\mathbf{x}},{t_0}),\varDelta ({\mathbf{x}},{t_1}), \ldots ,\varDelta ({\mathbf{x}},{t_{n - 1}})\} .$$

do not present a large variation, so fewer bits can be used to represent them. The exact number of bits required to represent *D*_*x*_ is given by:

$$\left\lceil {{{\log }_2}(\hbox{max} {V_x} - \hbox{min} {V_x} + 1)} \right\rceil .$$

Thus, *D*_*x*_ is stored by using

$$\left\lceil {{{\log }_2}(\hbox{max} {V_x} - \hbox{min} {V_x} + 1)} \right\rceil \times n$$

bits.

In a sequential processing unit, the voxels are compressed one by one, and the time required to compress a single voxel is proportional to *n* because *n* computations are required to determine *D*_*x*_, min *V*_*x*_, and max *V*_*x*_. Thus, when executed sequentially, the computational complexity of our algorithm is *m* × *n* where *m* is the number of voxels in the dataset.

However, the compression of all the voxels is independent, and consequently, it can be done in parallel. During the compression of a voxel, the computations to calculate *D*_*x*_ are independent, and they can also be parallelized. Moreover, when using parallel processing, min *V*_*x*_ and max *V*_*x*_ can be calculated in a time proportional to log _2_*n* through parallel reduction [6]. In a parallel implementation, the most expensive computations required by our algorithm correspond to finding min *V*_*x*_ and max *V*_*x*_. Consequently, in terms of computational complexity, our algorithm can compress a CTP dataset in a time proportional to log _2_*n* when running in parallel.

To retrieve the value of $I({\mathbf{x}},t)$, a sum needs to be performed: $C({\mathbf{x}}) + \varDelta ({\mathbf{x}},t)$. By using fixed size arrays to store $\varDelta ({\mathbf{x}},t)$ and $C({\mathbf{x}})$, $I({\mathbf{x}},t)$ can be retrieved in constant time. The data stored using less bits, which is $\varDelta ({\mathbf{x}},t)$, do not need to be modified. Thus, in our method, $I({\mathbf{x}},t)$ is determined using a single sum of values that can be retrieved in constant time.

### Implementation

The efficiency of our compression method is strongly dependent on the efficiency of the data structures used in its implementation, in particular for $C({\mathbf{x}})$ and $\varDelta ({\mathbf{x}},t)$.

$\varDelta ({\mathbf{x}},t)$ is an element of the set *D*_*x*_. All the elements in a set *D*_*x*_ are represented using the same number of bits. For instance, by considering the voxels at ${{\mathbf{x}}_a}$ and ${{\mathbf{x}}_b}$ in Fig. 2, six and eight bits are required to represent the elements in ${D_{x_a}}$ and ${D_{x_b}}$ respectively. Thus, because *n* = 24 in our datasets, ${D_{x_a}}$ requires 24 × 6 = 144 bits, and ${D_{x_b}}$ requires 24 × 8 = 192 bits to be represented. Because the amount of bits required to represent each *D*_*x*_ set varies, it is not possible to use a single fixed size array to store all the different *D*_*x*_ sets in memory.

Current computers are not capable of addressing memory blocks of an arbitrary amount of bits. Thus, all the *D*_*x*_ sets are contiguously stored in a fixed size array of 32 bits elements named D. A maximum of two elements from D need to be accessed to store and retrieve a particular $\varDelta ({\mathbf{x}},t)$ using a fixed amount of bit shift operations. The computational cost of these operations is constant, so they do not increase the computational complexity of reading and storing the values in D.

**Fig. 4:**
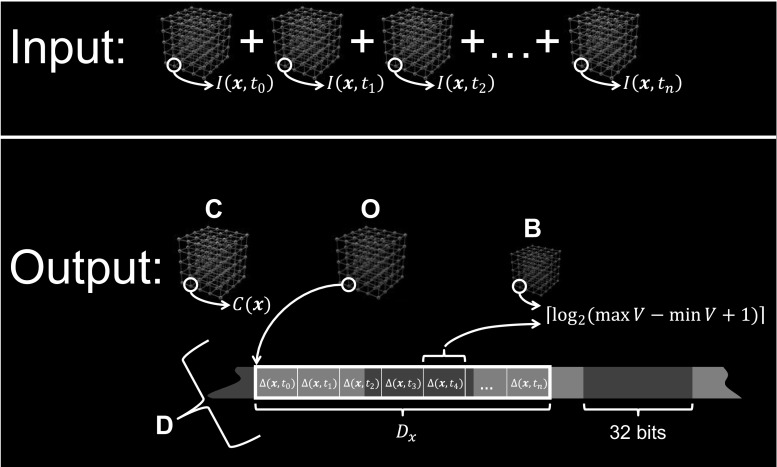
Data structures used in the implementation. B is a constant size array of 8-bit elements that stores the amount of bits used to encode the intensity values of a voxel. C is a constant size array of 16-bit elements used to store all the $C({\mathbf{x}})$ values. D is a constant size array of 32-bit elements used to store all the *D*_*x*_ sets. O is an offset to determine where a set *D*_*x*_ begins in the array D

An offset is provided to determine where a *D*_*x*_ begins in the array D. All the offsets are stored in a fixed size array of 32 bits elements named O. Another fixed size array of eight-bit elements, named B, is used to store how many bits are used to represent the elements in *D*_*x*_. In this way, different elements in *D*_*x*_ can be distinguished. The offsets can be quickly calculated by traversing B. However, O is provided to keep instant access to any *D*_*x*_ in D. Finally, an array of 16-bit elements, named C, is used to store all the $C({\mathbf{x}})$ values. The elements of C have 16 bits because they contain original intensity values from the 16-bit voxels. Figure 4 illustrates the data structures used in our implementation. The size of the resulting compressed data is the sum of the sizes of the arrays C, O, B, and D.

Three different implementations of our dynamic image compression for parallel processing (DICOPP) were developed:

- DICOPP CPU—a parallel implementation compressing the voxels using multiple threads in a many-core CPU and using a sequential method to calculate min *V*_*x*_ and max *V*_*x*_;
- DICOPP CPU PR—another parallel implementation targeted for a many-core CPU using multiple threads to calculate min *V*_*x*_, and max *V*_*x*_ through the parallel reduction method; and
- DICOPP GPU—a parallel implementation running on the GPU and calculating min *V*_*x*_ and max *V*_*x*_ sequentially.

During the implementation, we observed that using parallel reduction to calculate min *V*_*x*_ and max *V*_*x*_ on the GPU requires a more complex organization of the data in the GPU memory, which slows down the memory operations and results in an inefficient GPU implementation. For this reason, this alternative was abandoned. Also, only 24 values need to be evaluated to calculate min *V*_*x*_ and max *V*_*x*_, and at this scale, the benefits of using parallel reduction are not noticed.

Our implementations use the .NET framework version 4.0 [7] and C# [8] as programming language. These technologies were chosen because our implementations need to be integrated in an existing platform for medical image processing based on .NET. Our implementations use Fellow Oak DICOM (FO-DICOM) for .NET version 1.0.36 [9], which is a high-performance API for handling DICOM files. For the GPU computations, OpenCL 1.1 [10] was used. OpenCL is a framework for the development and execution of programs across platforms consisting of different types of processors such as CPUs, GPUs, digital signal processors, field-programmable gate arrays. OpenCL.NET version 2.2.9 [11] was used to integrate OpenCL with .NET. OpenCL.NET is a library that wraps the original OpenCL 1.1 API for .NET.

### Evaluation setup

All the compression techniques that are incorporated in the DICOM format were selected for comparison with our method. However, according to the DICOM specification, MPEG2 and MPEG-4 compressions are inherently lossy, and for this reason, they were excluded of our comparison. JPEG 2000 lossless was also excluded from our comparison because it is much slower than the other methods, without a considerable better compression ratio. Consequently, only the following techniques from the DICOM standard were used in our experiments:

- JPEG lossless, more precisely the JPEG process 14 (first-order horizontal prediction [selection value 1], DPCM, non-hierarchical with Huffman coding);
- JPEG LS lossless; and
- Run-length encoding (RLE).

Very efficient low-level implementations of the techniques from the DICOM standard were used in our comparison. For JPEG and JPEG 2000, an open-source C library named FreeImage [12] was used. Regarding JPEG LS, an open-source and optimized C++ library named CharLS [13] was used. Finally, for the RLE compression, the C++ implementation provided with the FO-DICOM library was used. Regarding our method, the three implementations described in Sect. 2.3 were used in our comparison.

All the selected techniques from the DICOM standard were used only to perform 2D compression, and as a result, they were used to independently compress all the slices in a CTP dataset. These techniques are not designed to be executed in massively parallel architectures. Thus, to provide a fair comparison of the compression time with our implementations, which were designed for these architectures, the compression of all slices were divided equally among the CPU threads available by a multithread application. In this manner, the thread overhead was minimized, and the usage of the CPU for the compression task was maximized. Regarding our method, the same approach was used in our CPU implementations, i.e., use all the available CPU threads and distribute load equally. In the GPU implementation, the compression time includes the time required by the transfers between the host application and the GPU device.

Ideally, GPU implementations of the other compression techniques should be used for the comparison. However, to the best of our knowledge, there is no GPU implementation available for these methods. For JPEG, there are many GPU-based codecs, but none of them presents the lossless compression mode.

To compare the time to access the decompressed data, intensities of all time steps of 320 voxels in 320 slices were retrieved sequentially in an application running on the CPU and accessing the compressed data in the host application memory. Our method does not require complete decompression of a CTP dataset, and in this manner, accessing the decompressed value of a single voxel is a straightforward way to compare the decompression performance of the evaluated methods. The compressed data produced by the three different implementations of our method are identical; therefore, reading time was computed only for one of the results.

To evaluate the impact of the number of processing units in the compression time of our method, the DICOPP CPU implementation was executed using from 1 up to 6 threads. The maximum of six threads was defined because this is the number of independent processing units available in the hardware configuration used (see Table 1).

**Table 1:** Hardware configuration used to execute the compression methods evaluated in our experiments

CPU name	Intel Xeon E5-2620
CPU clock	2.00 GHz
CPU cores	6
CPU threads	12
RAM memory	64 GB
GPU name	GeForce GTX TITAN
GPU driver version	331.65
GPU cores	2688
GPU clock	836 MHz
Dedicated video memory	6 GB GDDR5

The main goal of our compression technique is to enable faster transfer to cloud architectures. To evaluate this, the total transfer time of each compression method used in our comparison was computed. This time is calculated by adding: the compression time, the time to transfer the compressed data, and the time to read the compressed data. The time to transfer the compressed data was calculated by considering the theoretical transfer rate of the following network standards: OC-3/STM-1 [14], OC-12/STM-4 [14], 1000BASE-T [15], and OC-48/STM-16 [14], or 155, 622, 1000, and 2400 Mbps, respectively. 1000BASE-T is a standard for gigabit Ethernet networks. The other standards specify the transmission bandwidth for digital signals that can be carried on fiber-optic networks.

Our compression technique enables GPU processing directly from the compressed data. By processing the compressed CTP data, less data need to be transferred between host and GPU. This feature can speed up the total GPU processing time considerably because, in some applications, most of the time in a GPU computation is spent on data transfers. In order to evaluate the GPU processing time improvement, a GPU application that creates a mask from the CTP data was developed. The mask, which is defined by the double threshold 0–15 HU, is part of a noise reduction filter for dynamic CTP data described in [16]. In our evaluation, the developed GPU application computes this mask in two different ways: using the uncompressed data and using the compressed data generated by our method. In both ways, the time to compute the mask is measured including the time spent by the transfers between host and GPU.

All the evaluations described in this section were performed in the same hardware configuration (see Table 1) using Windows 7 Enterprise 64 bits as operating system. For all the time measurements, the high-resolution timing counters provided by the Win32 API were used.

## Results

Table 2 shows the performance results of the evaluated compression techniques applied to 20 CTP datasets described in Sect. 2.1. The DICOPP CPU PR implementation achieved a better compression time than the DICOPP CPU implementation in 85 % of the executions. As mentioned in Sect. 2.3, the CTP datasets time dimension is too short to substantially benefit from parallel reduction for computing min *V*_*x*_, and max *V*_*x*_.

**Table 2:** Compression time, reading time, and compression ratio for 20 datasets (mean ± SD [min., max.]) using different compression methods. The best results are underlined

Compression method	Compression time (ms)	Reading time (ms)	Compression ratio
JPEG LS	09911 ± 0398 [08879, 10806]	58267 ± 2546 [49924, 62052]	4.64 ± 0.29 [4.14, 5.55]
JPEG	14552 ± 0742 [12234, 16095]	43443 ± 1791 [37033, 44997]	2.09 ± 0.16 [2.74, 3.55]
RLE	09679 ± 0947 [08286, 11110]	15554 ± 0634 [13468, 16669]	2.31 ± 0.10 [2.12, 2.66]
DICOPP CPU	20350 ± 2602 [14157, 24239]	0.15 ± 0.36 [0, 1]	2.20 ± 0.17 [1.95, 2.75]
DICOPP CPU PR	17718 ± 1413 [14934, 20712]		
DICOPP GPU	05944 ± 0711 [04826, 07873]		

In our evaluation setup, all the data are transferred to CPU memory before being accessed or decompressed. Thus, all the reading and decompression operations are executed only in the host application. The reported time corresponds to the reading time of only 320 × 24 voxels, and not to the entire CTP dataset. Our method does not require full decompression of a dataset, and because of this, it achieved a read time many times lower than the best result from the other methods.

JPEG 2000 lossless took 132 and 470 s to compress and read the compressed data of a single CTP dataset. This is more than six times slower than the results in Table 2.

**Fig. 5:**
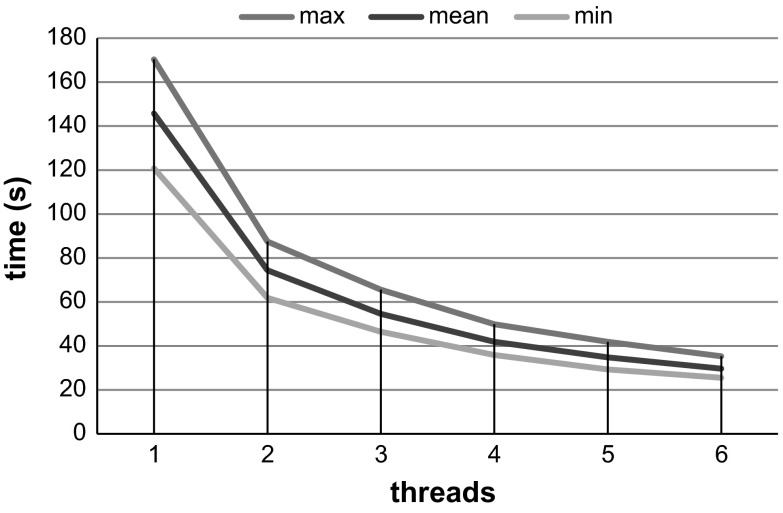
Maximum, mean, and minimum times (*vertical axis*) spent to compress 20 CTP datasets by using different number of threads (*horizontal axis*)

The number of processing units used to execute our compression method has a major impact in its compression time. To illustrate this, Fig. 5 shows the compression time obtained by using different number of threads for compressing 20 CTP datasets using the DICOPP CPU implementation. The standard deviations of the compression time of the executions using from 1 to 6 threads are, respectively, 14.27, 7.30, 5.76, 4.32, 3.83, and 2.86 s.

Table 3 shows the total transfer time (compression time + time to transfer compressed data + decompression time) for the 20 CTP datasets using the maximum transfer rate of four different types of network. As a reference, the first row of Table 3 shows the only the transfer time of an uncompressed dataset. DICOPP GPU achieved the lowest transfer time in all the network types listed in Table 3. However, in networks slower than the ones listed in Table 3, JPEG LS achieves a better transfer time because it has a better compression ratio. In faster networks, it takes longer to compress and transfer the data than to transfer the original data without compression.

**Table 3:** Total transfer time (in s) for 20 datasets compressed by different methods and using different network speeds (mean ± SD [min., max.])

	OC-3/STM-1 (s)	OC-12/STM-4 (s)	1000BASE-T (s)	OC-48/STM-16 (s)
Original Data	207.82	51.79	32.21	13.42
JPEG LS	113 ± 5.3 [096, 122]	79 ± 3.2 [68, 84]	75 ± 3.2 [64, 79]	71 ± 3.0 [61, 75]
JPEG	127 ± 5.4 [107, 134]	75 ± 3.0 [63, 78]	68 ± 2.7 [58, 71]	62 ± 2.5 [53, 64]
RLE	115 ± 4.7 [100, 123]	47 ± 1.9 [41, 50]	39 ± 1.6 [34, 41]	31 ± 1.4 [27, 33]
DICOPP CPU	115 ± 7.9 [089, 127]	44 ± 3.5 [32, 48]	35 ± 3.0 [25, 38]	26 ± 2.7 [19, 30]
DICOPP CPU PR	112 ± 7.4 [090, 123]	41 ± 2.5 [33, 45]	45] 32 ± 2.0 [26, 36]	36] 23 ± 1.6 [19, 27]
DICOPP GPU	100 ± 6.9 [080. 111]	29 ± 1.9 [23, 32]	20 ± 1.3 [16, 22]	12 ± 0.8 [09, 14]

The total transfer time is the sum of the compression time, time to transfer the compressed data, and the decompression time. The first row shows the transfer times of an uncompressed CTP dataset. The data transfer times were calculated based on the theoretical transfer rate of the network standards: OC-3/STM-1 [16], OC-12/STM-4 [16], 1000BASE-T [28], and OC-48/STM-16 [16]. Respectively, these transfer rates are: 155, 622, 1000, and 2400 Mbps. The best results are underlined

Regarding the GPU processing time, the GPU processing of the mask using the original and the compressed data took 2818 ± 382 [2664, 4392] and 1903 ± 186 [1712, 2668] milliseconds, respectively. Accordingly to these results, the GPU processing using the compressed data was, on average, more than 30 % faster than the processing of the original data.

## Discussion

The compression time of the DICOPP GPU implementation is notably faster than the other methods. Even simple algorithms, such as RLE running in parallel, are around 1.6 times slower than the DICOPP GPU implementation. Note that the implementations of our method used more abstraction layers than the other implementations used in the comparison. For instance, memory management in .NET applications is different from low-level applications, and this can result in a slower execution time when compared with C or C++ applications, which is the case of the other methods. However, despite the higher level of abstraction, the compression time of our CPU implementations is approximately only 5 to 10 s slower than the other methods.

As expected, our method presents negligible times to read the voxel intensities from the compressed data, as illustrated in Table 2. This is possible because our method is the only to provide direct access to the voxel intensities. The random access to voxel values has many advantages, and it enables the application of several imaging operations to the entire image data in the compressed form. Because of this direct access, operations such as local filtering and threshold-based segmentations can be performed without decompression. By doing this, our method saves memory (the compressed data are processed) and processing time (the decompression step is skipped). The direct access to voxel values provided only by our method also speeds up the GPU processing. This is possible because our method reduces the amount of data that need to be transferred between host application and GPU, which is also a common bottleneck in GPU-based computing. As presented in Sect. 3, the GPU computation of a mask from the CTP data was speeded up more than 30 % by using the compressed data produced by our method. We must highlight that exactly the same instructions were executed in the compressed and uncompressed representations of the CTP data. This speedup is only possible because of the direct access to the voxel values from the compressed data on the GPU.

As previously stated, the main goal in acute care is to provide fast results, and as observed in Table 3, the implementations of our method achieve better transfer times than all the others. In networks slower than 100 Mbps, our method was overcome by other compression methods. However, it is reasonable to assume that current cloud infrastructures provide connections with speeds that are higher than 100 Mbps. In fact, most of the current cloud providers offer direct connections up to 10 Gbps. For instance, Microsoft Azure [17] offers connections from 200 Mbps up to 10 Gbps, and Amazon Web Services [18] offers connections from 50 Mbps up to 10 Gbps. In these very fast connections, transferring the uncompressed data is faster than transferring the compressed data. However, these very fast connections are expensive and priced according to the offered speed. This means that our method enables a cost-effective usage of these connections. Our method can also reduce the GPU processing time of the CTP data. Because of this feature, our method not only contributes for a faster analysis of CTP data, which is crucial in acute stroke cases, but also to cheaper analysis on pay-per-use infrastructures. Thus, regarding the processing pipeline of CTP data on GPU-based cloud infrastructures, our compression method enables fast transfers and fast GPU processing, which consequently results in reducing costs and providing the faster image processing required when dealing with acute stroke patients.

Our compression technique was developed to be executed in massively parallel architectures. Thus, it is possible to achieve faster results when using more parallel processing units (see Fig. 5). Also, as observed in Table 3, our compression technique is the only one that enables reducing transfer times in fast data connections because of its fast compression and because it does not require a decompression step prior to processing. Because of these characteristics, our compression technique is better suited for future computational infrastructures than the other compression techniques evaluated, since it can benefit from massively parallel processing and fast data connections. We must emphasize that, if ignoring the cost aspect, there are connections speeds currently available that are fast enough to be used for transferring uncompressed CTP data. However, with more powerful parallel processing devices, our method can become beneficial even with these connection speeds. Thus, because of these trends, we believe that our method is beneficial not only in current cloud infrastructures but also in the upcoming cloud infrastructures.

The compression ratio of our method is inferior to the compression ratio of the other methods. To improve our compression ratio, different preprocessing operations could be applied. However, this preprocessing can make the execution of our compression technique considerable longer. To avoid that, the CTP processing pipeline has to be carefully analyzed to identify whether the adoption of preprocessing steps will effectively result in a faster data transfer, which is the main goal of our work. For instance, usually the CTP analysis requires the application of a noise reduction filter. In a new pipeline configuration, this noise reduction can be done before the compression in order to achieve a better compression ratio. Noise reduction may also improve the compression ratio of our method because noise strongly influences the variation of the voxel values over time. It is expected that thick slices have less noise, and it may result in better compression ratios. A detailed study to assess the effects of different noise levels in the performance of our compression method can be performed. However, in this paper, we focused on the evaluation of our compression method in the image data that are generated in clinical practice.

Apart from noise, motion artifacts can also affect the compression ratio of our method. Again, a possible solution is a preprocessing step for motion correction before the compression step [19]. However, this will result in increasing processing time. We evaluated our method in actual patient data, which included motion artifacts, and as shown in Fig. 3, the effects of motion do not have a strong impact on our compression ratio. Motion does not affect the compression ratio of our method considerably because, in different time frames, different types of tissue rarely overlap, and thus constant geometrical locations still have similar intensity values. The only exceptions are the areas around the skull, which are a small portion of the image data. However, even in these areas, the amount of bits required to represent the compressed data are still considerably smaller than the original amount of bits used in the uncompressed data.

Perhaps, the most effective preprocessing step that could be applied is a simple threshold segmentation and removal of useless data (i.e., the air around the patient). Nevertheless, we focused in evaluating our method in original patient data. An extensive analysis of the different techniques that can be combined with our compression method was beyond the scope of this study.

Our goal was to provide a compression technique to be used in a specific clinical practice rather than to be used as a general compression technique. In clinical practice, we are dealing with large datasets that are very precisely defined (±24 time steps of approximately 320 slices of 512 × 512 pixels of 16 bits) and that are well accepted worldwide. Since CTP acquisitions are performed tens of thousands times per year, we believe that a specific and applied compression technique is worth studying. Although our technique is applied to and focused on CTP data, we believe that any other medical image time series could be potentially suited for compression by our algorithm. For example, all the medical images used in the experiments described in [2] have the necessary characteristics to be exploited by our compression algorithm, which is a small variation of voxel values over time.

### Related work

Previous works also explored the redundancies in the temporal dimension of medical image data for compression purposes. The work presented in [20] calculates the differences between two contiguous images from a medical image time series and store these differences using eight bits when this is possible. When this difference cannot be expressed using eight bits, the original 16 bits are used. Because of this approach, the theoretical maximum compression ratio achieved by this method is 2. In this method, to retrieve the intensities from a particular time step, it is necessary to decompress all intensities from the previous time steps. The main differences between this and our method are: our method achieves compression ratios greater than 2, and in our technique, any arbitrary image intensity in the four-dimensional space can be retrieved independently with a constant computational complexity.

Other compression techniques explore the effect of motion in 4D medical images. Motion is a feature especially present in 4D cardiac images. In the context of exploring motion for compression purposes, [21] proposed a technique based on the combination of a predictive image compression and a motion compensation technique. The work presented in [22] evaluates the motion in 4D medical images for compression purposes using motion fields that produce input parameters for a neural network used for motion estimation. [22] combines motion analysis with segmentation, block matching, and expert knowledge, to develop a framework for 4D medical compression. The authors of [23] apply recursively a multiframe motion compensation process that employs 4D search, variable block-sizes, and bidirectional prediction for reducing redundancies in spatial and temporal dimensions. All these three techniques were developed for achieving high compression ratios, and because of their complexity of compression and decompression, they are not well suited for the fast processing as required in acute care situations. Also, differently from our technique, they require a decompression step before processing.

Another common approach is to adapt or use existing sound, image, or video compression techniques for 4D medical image data. However, most of these compression techniques, like MPEG-2 and MPEG-4, are lossy and, for this reason, cannot be used in the same context as the proposed technique. Regarding lossless compression, the authors of [24] proposed a technique for 4D medical images based on the H.264/AVC standard for video compression. Again, this compression technique was designed to achieve high compression ratios, being too complex for producing fast response.

In CTP data, any particular voxel can be considered as an independent time series. Time series compression techniques can be applied independently for each voxel. However, most time series compression techniques are fundamentally lossy [25] and consequently cannot be used for the purposes of this study.

Regarding the lossless compression of time series, current techniques focus on the compression of long time series and are based on very complex models [26–29] that may even require the usage of a database for prediction purposes [28]. Because these techniques are developed for compressing long time series, it is not feasible to use them in CTP datasets, which present only 24 time steps. To illustrate this problem, the smaller model mentioned in [29] requires 192 bits only to store the initial conditions of the model equations describing a time series. This represents half of the size of entire time series of a particular voxel in CTP datasets (24 × 16 bits). The lossless time series compression can be also based on features that are not available in CTP datasets, such as multichannel [30] or multispectral information [31]. In short, the usage of state-of-the-art lossless time series compression in the time series from CTP datasets would not be effective because of the short length of these time series.

## Conclusion

In this paper, we presented a new method to compress CTP data that take advantage of data redundancy in the time dimension. The proposed algorithm reduces the image size by using fewer bits to represent data that do not vary much along time. This method focuses on providing faster transfer of CTP data to GPU-based cloud infrastructures; therefore, a balance between compression ratio and compression time has been pursued, which is different from many compression methods which pursue good compression ratios. Our algorithm was designed for massively parallel architectures, and it is well suited for many-core CPU or GPU execution.

The proposed method was applied to 20 datasets and obtained the faster results compared to the lossless compression techniques adopted in the DICOM standard, despite its inferior compression ratio.

The resulting data representation offers direct random access for subsequent GPU processing, which is a feature not found in the other compression methods. Because of this, our time for retrieving information from the compressed data is negligible. This feature also makes it possible to reduce the time to transfer CTP data between host application and GPU because only the compressed form of the CTP data needs to be used in these transfers. Consequently, the GPU processing of CTP data can be speeded up when using the data in compressed form.

Currently, different ways to improve the compression ratio of our method are being investigated. This investigation focuses on the usage of fast techniques for noise reduction, motion identification, and segmentation of meaningless image elements. All these techniques need to be compatible with current clinical practices adopted when analyzing CTP data.
